# JAX-RNAfold: scalable differentiable folding

**DOI:** 10.1093/bioinformatics/btaf203

**Published:** 2025-04-25

**Authors:** Ryan K Krueger, Max Ward

**Affiliations:** School of Engineering and Applied Sciences, Harvard University, Cambridge, MA 02138, United States; Department of Computer Science and Software Engineering, The University of Western Australia, Crawley, WA 6009, Australia

## Abstract

**Summary:**

Differentiable folding is an emerging paradigm for RNA design in which a probabilistic sequence representation is optimized via gradient descent. However, given the significant memory overhead of differentiating the expected partition function over all RNA sequences, the existing proof-of-concept algorithm only scales to ≤50 nucleotides. We present JAX-RNAfold, an open-source software package for our drastically improved differentiable folding algorithm that scales to 1,250 nucleotides on a single GPU. Our software permits the natural inclusion of differentiable folding as a module in larger deep learning pipelines, as well as complex RNA design procedures such as mRNA design with flexible objective functions.

**Availability and implementation:**

JAX-RNAfold is hosted on GitHub (https://github.com/rkruegs123/jax-rnafold) and can be installed locally as a Python package. All source code is also archived on Zenodo (https://doi.org/10.5281/zenodo.15003072).

## 1 Introduction

Ribonucleic acid (RNA) is a fundamental molecule in any biological organism and is therefore an attractive substrate for new therapeutic agents (Kole *et al.* 2012; Wang *et al.* 2020; Damase *et al.* 2021; Zhu *et al.* 2022). Despite the advent of high-throughput sequencing and synthesis technology, experiments at the bench are costly and time-consuming. Therefore, computational modeling is used to obtain rapid approximations to experimentally determined structures (Zuker 2003; Reuter and Mathews 2010; Lorenz *et al.* 2011; Rivas 2020; Sato and Hamada 2023). Accurate computational prediction of RNA structure from only the sequence is a widely studied problem (Zuker and Stiegler 1981; Mathews 2006; Rivas 2013; Szikszai *et al.* 2022). The inverse problem is equally important, but appears to be much harder. Instead of predicting the structural properties given the sequence, many biomedical applications require finding a sequence that has given structural properties. This is generally called an “inverse” or “backwards” problem. In the context of RNA, this problem is known as the “RNA design” problem.

**Figure 1.**
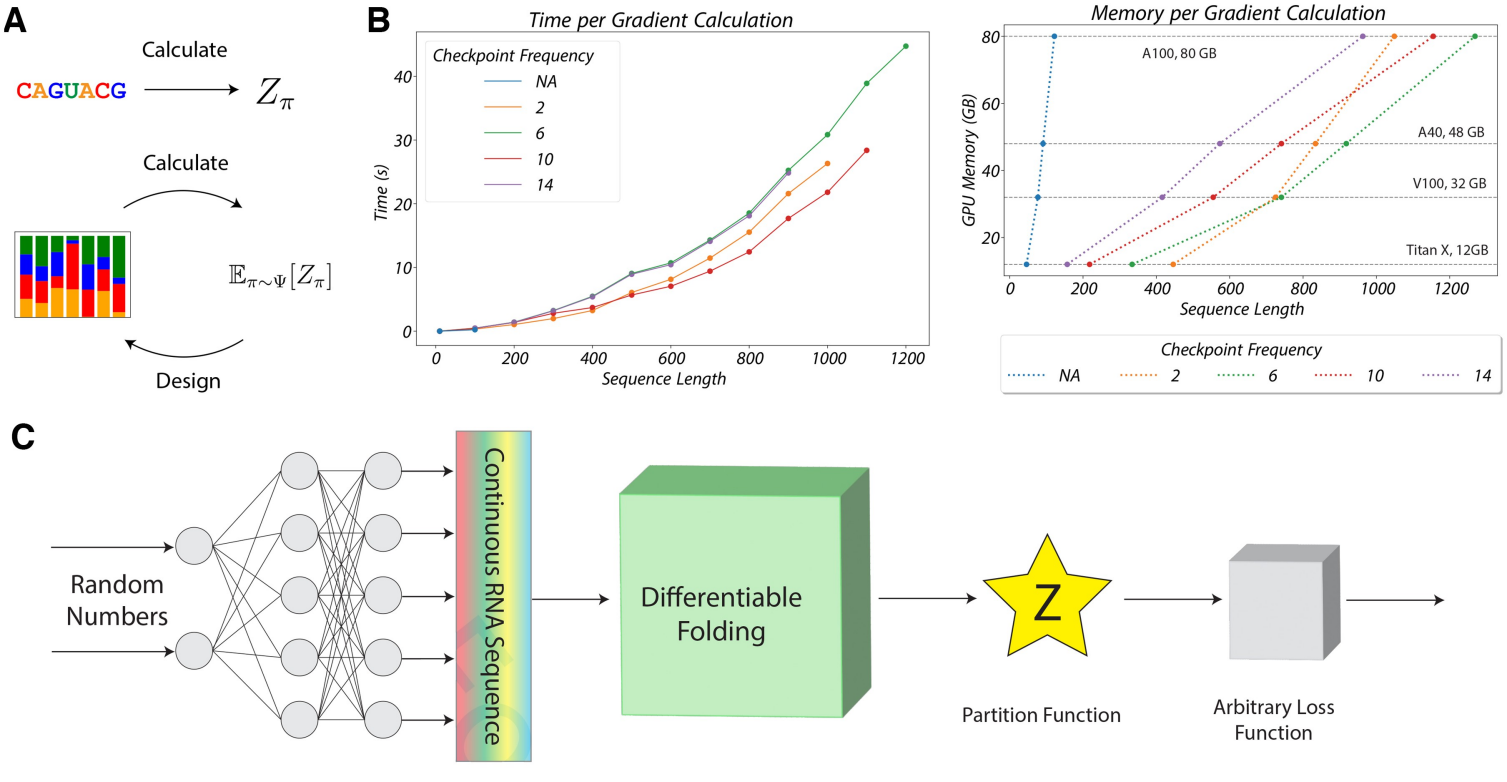
An overview of our improved algorithm for differentiable folding. **A**. RNA folding compared to differentiable RNA folding. In traditional RNA folding, McCaskill’s algorithm computes the partition function of a discrete RNA sequence. Under differentiable folding, the generalized McCaskill’s algorithm computes the expected partition function of a distribution of RNA sequences. When implemented in an automatic differentiation framework, this distribution can be optimized via gradient descent. **B**. The time and memory cost with respect to sequence length for our optimized differentiable folding algorithm. On the left, we plot the time per gradient evaluation as a function of sequence length for different checkpointing frequencies. On the right, we plot the memory cost per gradient evaluation as a function of sequence lengths for the same set of checkpointing frequencies. **C**. An overview of our method to train a generative model that produces a probabilistic sequence that minimizes a continuous loss function based on the partition function. We overparameterize the optimization problem by training a generative network to produce a sequence distribution that minimize the objective function. Once trained, discrete sequences can be sampled from the predicted sequence distribution.

Recently, Matthies *et al.* (2024) developed a differentiable RNA folding algorithm for RNA design. Intuitively, this approach inverts McCaskill’s famous algorithm for computing the RNA partition function, defined as


(1) $$Z_{\pi }=\sum_{s\in S}e^{-\beta E(s|\pi )}$$

where *S* is the set of all possible structures, β is a thermodynamic scaling factor, and E(s|π) is the free energy of the structure *s* with respect to the sequence π (McCaskill 1990). Matthies *et al.* achieve this inversion by generalizing this algorithm to efficiently compute the expected partition function over a probabilistic sequence representation,


(2) $$Z_{\Psi }=E_{\pi ∼\Psi }[Z_{\pi }]$$

where Ψ denotes a distribution of sequences and P(π|Ψ) denotes the probability of a sequence sampled from the distribution. When implemented in an automatic differentiation framework, Ψ (represented as the parameter of a product of categorical distributions over nucleotides) can be optimized via gradient descent (Fig. 1A). While Matthies *et al.* demonstrated the algorithm for simple cases of structure design, the original proof-of-concept implementation is limited to sequences up to 50 nucleotides. See the Supplementary data for a complete treatment of differentiable folding.

We present a drastically improved algorithm for differentiable folding and implement our method in an open-source software package, JAX-RNAfold. Our algorithm permits gradient calculation for sequences up to n=1,250 nucleotides in length (Fig. 1B) because the memory usage at n=1,250 is reduced by a factor of 15, 000. In addition, we implement support for the treatment of differentiable folding as a module in a larger deep learning pipeline (Fig. 1C). We demonstrate the use of our software for both structural design, as overparameterization via a fully connected neural network improves upon vanilla optimization, and mRNA design, as support for longer sequences inspires a formulation of mRNA design as a continuous optimization problem.

## 2 Materials and methods

In this section, we briefly describe a suite of improvements to scale the algorithm of Matthies *et al.* (2024) to longer sequences. We then describe an example application of differentiable folding as a module in a larger (generative) deep learning pipeline, and our formulation of the mRNA design problem for optimizing coding sequences via differentiable folding.

### 2.1 Algorithmic improvements

The original implementation of differentiable folding was limited to sequences of up to 50 nucleotides in length (Matthies *et al.* 2024). The primary bottleneck for this algorithm was memory usage, and GPUs have limited memory. Improvements to the algorithm were necessary to scale differentiable folding to experimentally relevant lengths. Note that the original McCaskill’s algorithm (McCaskill 1990) required $O(n^{2})$ memory for a sequence of *n* nucleotides, which is relatively frugal. However, continuous and differentiable folding first adds a large constant factor to deal with the continuous sequence, and also uses $O(n^{3})$ memory since intermediate values are stored for backpropagation (Matthies *et al.* 2024). We developed a suite of algorithmic optimizations that drastically reduce the space and time requirements for differentiable folding.

The optimizations can be roughly split into three categories: better recursions, checkpointing, and numerical issues. The *better recursions* category includes improving the dynamic programming recursions used to compute the partition function. These all lead to constant factor improvements to speed and memory usage, although this constant factor was sometimes large. The treatment of coaxial stacks, terminal mismatches, dangling ends, and multiloop branches was changed, which saved a factor of 256. In addition, internal loops were also optimized saving a factor of 256.

We used *checkpointing* to exploit the time-memory tradeoff inherent to backpropagation. Gradient calculation by backpropagation entails saving intermediate values so that gradients can be calculated. Instead of storing all intermediate values, one can recompute them from checkpoints. We found that a judicious use of this could reduce our theoretical memory usage asymptotically from $O(n^{3})$ to $O(n^{2.5})$.

Improving *numerical issues* was the final step. With the algorithm able to handle longer lengths, numerical issues arose in the calculation. GPUs can generally only handle up to 64 bits of floating point precision. The dynamic programming algorithm takes a weighted sum over the partition functions of all $4^{n}$ sequences and each partition function is a sum of an exponential number of structures (Stein and Waterman 1979). This leads to very large values that can overflow. There are two methods generally used to mitigate such overflow: (i) computing values in log-space (used in RNAstructure (Reuter and Mathews 2010)) or (ii) per-nucleotide scaling (used in ViennaRNA (Lorenz *et al.* 2011)). We found that log-space is problematic for gradient calculations, because addition in log-space must be approximated, but the gradient of the approximation is not necessarily an approximation of the gradient. To avoid this issue, we chose to use per-nucleotide scaling.

### 2.2 Generative deep learning method

In its basic form, differentiable folding directly optimizes a continuous RNA sequence via gradient descent. However, as demonstrated in Matthies *et al.* (2024), vanilla gradient descent does not always find optimal solutions for structural RNA design.

One popular solution for navigating non-convex landscapes is overparameterization (Hoyer *et al.* 2019; Both *et al.* 2023). Rather than optimizing over raw parameters, one can optimize over the parameters of a neural network whose outputs are the parameters that one wishes to optimize. Meanwhile, a defining advantage of differentiable folding is that it is gradient based and therefore can be used as a module in a larger deep learning pipeline.

We devised a simple generative deep learning approach to improve upon the vanilla gradient descent performed in Matthies *et al.* (2024). For a given RNA design problem, rather than optimizing over the continuous sequence itself, we optimize over the parameters of a fully connected neural network that outputs candidate continuous sequences as input to our folding algorithm. The input to the network is a fixed random seed. See Fig. 1C for an overview. Crucially, observe that such end-to-end training is only possible because the partition function calculation is itself differentiable; otherwise, we would need a large training set to learn from, which does not readily exist for RNA design.

### 2.3 mRNA design via continuous optimization

Matthies *et al.* (2024) demonstrated the application of differentiable folding to structure design. Given our improved algorithm scaled to experimentally-relevant lengths, we sought to formulate the mRNA design problem as a continuous optimization problem amenable to differentiable folding.

There are many objectives one may want to optimize for mRNA design. For our purposes, we use a simple but widely accepted formalization. This simplifies the problem to optimizing two objectives simultaneously: the stability of the sequence and the Codon Adaptation Index (CAI) (Sharp and Li 1987). We refer to this as “stability-CAI optimization.” Implementing this formulation of the mRNA design problem involves two technical challenges. First, CAI is not defined for continuous sequences. We define the *expected CAI* for a distribution of sequences Ψ and provide an algorithm for efficiently calculating this quantity. Second, the expected partition function calculation operates at the nucleotide level but an optimized mRNA sequence must code for the target protein. We account for this constraint by defining an additional term in our loss function describing the probability that a sequence sampled from Ψ codes for the target protein. See the Supplementary data for a generic formulation of stability-CAI optimization.

While we focus on stability-CAI optimization, our software permits significant flexibility in objective function formulation. For example, one could optimize for target dicodon frequencies, structural motifs, or properties of untranslated regions.

## 3 Results

In this section, we evaluate the scaling of our improved algorithm to longer sequences. We then demonstrate two candidate applications of this algorithm: (i) structural design via overparameterization and (ii) mRNA design. See Supplementary Table S3 for the total wall clock time required for each optimization.

### 3.1 Improved scaling

We sought to evaluate the scaling of our algorithm for gradient calculation in both space and time on a single GPU. We first evaluated the memory scaling for a range of checkpointing frequencies by measuring the maximum possible length for gradient calculation on a set of GPU models with varying memory capacity. We then evaluated the time per gradient calculation as a function of sequence length for a range of checkpointing frequencies on an 80 GB NVIDIA A100 GPU. Our results are depicted in Fig. 1B. Taken together, our suite of algorithmic improvements permits efficient gradient calculations for sequences of up to 1, 250 nucleotides on a single GPU. Given that the memory of the original differentiable folding algorithm scales as $O(n^{3})$, this represents a decrease in memory by >15,000× at n=1,250.

### 3.2 Example: Structural optimizations

Matthies *et al.* (2024) demonstrated the proof-of-concept differentiable folding algorithm for structural design on all Eterna100 sequence/structure pairs with ≤50 n.t. The Eterna100 dataset (Anderson-Lee *et al.* 2016; Koodli *et al.* 2021), a standard benchmark for RNA secondary structure design (Portela 2018; Shi *et al.* 2018; Koodli *et al.* 2019, 2021; Rubio-Largo *et al.* 2024; Wirecki *et al.* 2025), was chosen for its diverse range of structures and well-characterized challenges, including difficult-to-design motifs such as short stems, bulges, and multiloops. In addition to significant improvements in computational cost, our software extends this vanilla optimization method by permitting the treatment of differentiable folding as a module in a larger deep learning pipeline. To demonstrate this strength, we employed the generative deep learning method described above for structural optimization on a subset of the Eterna100 dataset. We define the objective function as described in Matthies *et al.* (2024) (see Supplementary data). Simple overparameterization with a neural network drastically improves optimization for those puzzles considered in Matthies *et al.* (2024) for which the probability of the designed solution folding into the target structure either (i) is lower than at least one of the corresponding probabilities for the provided answers or (ii) has low absolute value (see Table 1). For a fair comparison with Matthies *et al.* (2024), we applied terminal mismatches and dangling ends in accordance with their default treatment in the current version (2.7.0) of ViennaRNA (Lorenz *et al.* 2011). Nearest Neighbor parameters were also chosen to match the current version of ViennaRNA. The Eterna100 was designed using an older version of ViennaRNA (1.8.5), so some puzzles may not be solvable with these more recent parameters. Regardless, it serves as a widely accepted benchmark for comparison.

**Table 1.** Performance of our overparameterized optimization versus the vanilla optimization in Matthies *et al.* (2024) on a subset of Eterna100 puzzles. Performance is measured as the probability of the sequence folding into the target structure. “Neural Network” and “Original” correspond to sequences optimized via our method in which we overparameterize sequence space with a neural network and via the optimization directly over sequence space in Matthies *et al.* (2024), respectively. “Answer 1” and “Answer 2” correspond to the provided solutions in the Eterna100 dataset. For each puzzle, the underlined value represents the solution with the highest probability.

Puzzle ID	Neural Net	Original	Answer 1	Answer 2
15	0.416	0.002	0.403	0.540
20	0.610	0.209	0.244	0.588
41	0.407	0.021	0.001	$\approx 10^{-6}$
57	$\approx 5.5^{-7}$	$\approx 10^{-8}$	$\approx 10^{-12}$	$\approx 10^{-14}$
65	0.351	0.101	0.133	0.136
66	0.006	0.003	0.001	$\approx 10^{-4}$
88	0.026	0.011	0.014	0.013

To further demonstrate the utility of overparameterization, we performed structural optimization for the entire Eterna100 dataset using our default recursions (see Supplementary Table S1). Overparameterization yields improved optimization for 85 of 100 puzzles. We next sought to evaluate the extent to which our generative approach can produce solutions beyond that of a trivial design algorithm, i.e. setting paired positions to G and C and unpaired positions to A. Across all 100 puzzles, our method on average produces more GC/CG pairs (90.3% vs 59.4% and 59.3%) and less unpaired A’s (65.1% vs 80.6% and 79.2%) than the two reference solutions in the Eterna100. Crucially, our method can find solutions with a higher probability yet less agreement with the trivial GCA-solution compared to the Eterna100 solutions. For example, our method’s solution for Puzzle 6 has a probability of $1.81\times 10^{-8}$ vs $2.00\times 10^{-12}$ compared to one provided solution, yet lower proportions of GC/CG pairs and unpaired A’s by 8.7% and 39.9%, respectively (Supplementary Fig. S1). See Supplementary Fig. S1 for two additional examples.

### 3.3 Example: mRNA optimizations

As a second demonstration of our method, we designed coding sequences for five benchmark protein sequences—four sequences from Wayment-Steele *et al.* (2021) as well as Mini-GFP—via our formulation of stability-CAI optimization. For each protein sequence, we considered two optimization problems: (i) unconstrained CAI and (ii) CAI≥0.8. We seeded our algorithm with a continuous embedding of the LinearDesign solution (Zhang *et al.* 2023). Our algorithm consistently improved the sequence, yielding values of ΔΔG up to −1.11 kcal/mol. For each sequence, we only ran our method once. It should be observed that multiple runs can lead to better results. See Table 2 for these results and the Supplementary data for complete implementation details.

**Table 2.** Optimized performance of five benchmark mRNA sequences via our method and LinearDesign (Zhang *et al.* 2023). Performance is measured as the ensemble free energy of the sequence, defined as $-kT\;\text{log}\;\left(Z_{\pi }\right)$. All values are reported in kcal/mol. For each sequence, we considered two optimization targets: stability optimization (i) with unrestricted CAI and (ii) with CAI≥0.8. The LinearDesign sequence was used as input to our method for each optimization run.

	Unconstrained		**CAI** ≥0.8	
	LinearDesign	Our Method	LinearDesign	Our Method
**MEV**	−114.84	−114.92	−112.96	−113.04
**Mini-GFP**	−207.65	−208.59	−205.15	−205.15
**Nanoluciferase**	−452.34	−452.38	−451.29	−452.01
**spike RBD**	−411.55	−412.59	−407.50	−408.61
**eGFP + degron**	−546.92	−547.71	−546.56	−547.17

One strength of our method highlighted by these example optimizations is its ability to consistently improve sequences in a single restart. This can be compared to the method of Dai *et al.* (2024) which requires a batch of random initializations and can yield worse values of ΔG than the initial sequence, whereas our method only requires a single initialization and will never make ΔG worse. One notable optimization is eGFP with CAI≥0.8, for which our method found a sequence with lower EFE than the LinearDesign solution with unrestricted CAI.

We found that overparameterization did not affect our results. That is, we achieved the same result after removing the neural network. This is in contrast to structural optimization where it is essential. This appears to be because both end up in the same local optima due to the strong prior provided by starting optimization with LinearDesign’s output.

## 4 Discussion and conclusion

Differentiable folding presents a promising avenue toward next-generation RNA design—not only can it efficiently navigate sequence space for arbitrary objective functions, but it can be combined with deep learning methods for superior performance. The high memory cost of differentiation presents the largest barrier for various practical applications. We present a drastically optimized differentiable folding algorithm that achieves a 25× increase in sequence length on a single GPU. We also, for the first time, integrate this physics-based calculation in a larger end-to-end deep learning pipeline for efficient search. Crucially, this integration differs from typical deep learning approaches in that it does not require a pre-collected training dataset, instead treating the search problem as a self-supervised learning task with an infinite, fully differentiable data source. We demonstrate the strength of our software via improved structural optimizations, and a novel mRNA design formulation that improves upon state-of-the-art.

There is significant opportunity for future work in both scaling and applications. Regarding scaling, this includes (i) distribution across multiple GPUs, (ii) using one of several simplified models of RNA thermodynamics that achieve comparable accuracy, and (iii) devising a GPU-compatible linearized version of the algorithm akin to LinearPartition, which efficiently approximates the partition function via beam-pruning. However, it is possible that not including low-probability structures in the partition function calculation would degrade the quality of the gradient signal, and implementing this algorithm on a GPU is non-trivial. Dai *et al.* (2024) have already implemented the linearized partition function on CPU and found some preliminary results. Their findings are discussed further in the Supplementary data.

Regarding applications, there is significant opportunity to devise both alternative optimization functions and improved deep learning architectures. While not explored in this work, differentiable folding is amenable to complex mRNA design strategies, such as incorporating UTRs and imposing structure constraints on subsequences. This flexibility permits not only design with respect to existing metrics, but also the development of new metrics for mRNA stability and therefore a deeper understanding of mRNA thermodynamics. Likewise, there is also opportunity to improve our search algorithm beyond a simple overparameterization. Rather than training the weights of the network from scratch for each target mRNA, such a network could be pretrained to produce an optimized initial continuous sequences for a target mRNA.

Our algorithm is available for download at https://github.com/rkruegs123/jax-rnafold. The flexibility of differentiable folding combined with its natural integration with deep learning promises to offer improved *de novo* design methods as well as a deeper understanding of RNA thermodynamics.

## Supplementary Material

btaf203_Supplementary_Data

## Data Availability

JAX-RNAfold is hosted on GitHub (https://github.com/rkruegs123/jax-rnafold) and can be installed locally as a Python package. All source code is also archived on Zenodo (https://doi.org/10.5281/zenodo.15003072).
