# Supplementary material for: JAX-RNAfold: scalable differentiable folding
Source: Bioinformatics. 2025 Apr 25;41(5):btaf203. doi: 10.1093/bioinformatics/btaf203 (PMC12064173; doi:10.1093/bioinformatics/btaf203)
Supplement: btaf203_Supplementary_Data [file btaf203_supplementary_data.pdf]

Differentiable Partition Function

Matthies et al. [2023] introduce the notion of a differentiable partition function and give a polynomial time algorithm to calculate it. This partition function differs from Equation 1 in two important ways. First, it is defined for a distribution of sequences Ψ.

$$\mathcal{Z}_\Psi = \sum_{\pi\in\Psi}\sum_{s\in S} e^{-\beta E(s|\pi)} P(\pi|\Psi) \tag{S1}$$

We use $P(\pi|\Psi)$ to denote the probability of a sequence sampled from the distribution. In short, $\mathcal{Z}_\Psi$ is an expected partition function: $\mathcal{Z}_\Psi = \mathbb{E}_{\pi\sim\Psi}[Z_\pi]$.

It should be observed that the algorithm presented in Matthies et al. [2023] only handles Ψ constructed from a set of independent categorical distributions for each nucleotide. For a sequence of length n,

$$\Psi \in [0,1]^{4\times n}, \text{where } \sum_{i=1}^{4} \Psi_{i,j} = 1 \tag{S2}$$

Let $\pi_j \in [0,4)$ be an integer denoting the nucleotide identity of the jth nucleotide in the sequence π. Then,

$$P(\pi|\Psi) = \prod_{j=1}^{n} \Psi_{\pi_j,j} \tag{S3}$$

The second important difference between the work of Matthies et al. [2023] and McCaskill's partition function [McCaskill, 1990] is that the algorithm to compute $\mathcal{Z}_\Psi$ is differentiable. This means that gradients can be taken with respect to the input, Ψ. So, we can define a continuous and differentiable loss function over $\mathcal{Z}_\Psi$, $\mathcal{L}(\mathcal{Z}_\Psi)$, and optimize Ψ via gradient descent.

Structure Design

To design a sequence that folds into a target secondary structure, we follow the optimization formalism introduced in Matthies et al. [2023]. Given a target secondary structure s, we define the same objective function as in Matthies et al. [2023], i.e.

$$\mathcal{L}_s(\Psi) = -\log\left(\frac{Z_{s,\Psi}}{Z_\Psi}\right) \tag{S4}$$

where

$$Z_{s,\Psi} = \mathbb{E}_{\pi\sim\Psi}[e^{-\beta E(s|\pi)}] \tag{S5}$$

In Matthies et al. [2023], $Z_{s,\Psi}$ is referred to as the *sequence partition function* and can be computed efficiently via differentiable folding by restricting the permissible paired and unpaired bases in the calculation of Z_Ψ. Note that when Ψ is a one-hot encoded vector (corresponding to a discrete sequence), $\frac{Z_{s,\Psi}}{Z_\Psi}$ reduces to

$$\mathbb{E}_{\pi\sim\Psi}[p(s|\pi)] = \mathbb{E}_{\pi\sim\Psi}[\frac{e^{-\beta E(s|\pi)}}{Z_\pi}] \tag{S6}$$

but in general

$$\frac{Z_{s,\Psi}}{Z_\Psi} \leq \mathbb{E}_{\pi\sim\Psi}[p(s|\pi)] \tag{S7}$$

via Jensen's inequality. The derivation of this appears in the first version of Dai et al. [2024], but not in the later version. We find in practice that the approximation is close and the error quickly drops to zero as we approach a one-hot solution.

mRNA Design via Stability-CAI Optimization

In this section we provide details of our mRNA design procedure via stability-CAI optimization, and place our work in the context of existing mRNA design literature.

Optimization Problem Formulation

To design an optimal coding sequence for a target protein sequence, we optimize two objectives simultaneously: the stability of the sequence and the Codon Adaptation Index (CAI) [Sharp and Li, 1987]. We give a generic formulation of this "stability-CAI optimization:"

$$\Omega(\pi|\alpha) = f(s(\pi), \text{CAI}(\pi|\alpha)) \tag{S8}$$

where $\Omega(\pi|\alpha)$ is the objective function with an amino acid sequence α a potential mRNA sequence π. The function $s(\pi)$ measures the stability of the sequence and $\text{CAI}(\pi|\alpha)$ is the CAI of the RNA sequence given amino acid sequence. The function f is used to combine the stability and the CAI.

First, we consider the stability function $s(\pi)$. For a sequence π, the ensemble free energy (EFE) is related the partition function defined in Equation 1 via

$$\text{EFE}(\pi) = -\frac{1}{\beta}\ln(Z_\pi) \tag{S9}$$

Minimizing EFE is generally understood to be a proxy for maximizing stability. We can therefore use the generalized form of Z_π defined in Equation S1 as a proxy for stability, allowing us to optimize for sequences with high Z_π.

Second, we consider the contribution of $\text{CAI}(\pi|\alpha)$. Given a reference set of genes, CAI is computed as a a geometric mean

$$\text{CAI}(\pi|\alpha) = \left(\prod_{1}^{L}\frac{f_i}{\max(f_i)}\right)^{\frac{1}{L}} \tag{S10}$$

where $L = |\alpha| = \frac{|\pi|}{3}$, f_i denotes the frequency of the ith codon encoded in π, and $\max(f_i)$ denotes the maximum frequency over all possible codons for the ith amino acid in α. For a discrete RNA sequence π, CAI captures the codon usage bias for a given organism. We define the *expected CAI* for a distribution of sequences Ψ,

$$\text{ECAI}(\Psi) = \sum_{\pi} P(\pi|\Psi)\cdot\text{CAI}(\pi|\alpha_\pi) \tag{S11}$$

where $\text{CAI}(\pi|\alpha_\pi)$ is defined as in Equation S10 and α_π is the amino acid sequence corresponding to π.

Note that we do not restrict the calculation of $\text{ECAI}(\pi)$ to the target protein sequence α as this would underapproximate the CAI of the distribution of sequences. Instead, we compute an expectation over all codon sequences regardless of if they are valid for α. Importantly, $\text{ECAI}(\Psi)$ can be computed efficiently by computing the contribution of each probabilistic codon independently.

The constraint that the nucleotide sequence must code for the target protein imposes an additional challenge for differentiable folding as the dynamic programming recursions are defined at the nucleotide level, rather than at the codon level. To control for this, we define an additional term describing the probability that a sequence sampled from Ψ

codes for the target protein α:

$$P(\alpha|\Psi) = \prod_{i=1}^{L} P(\alpha_i|\Psi) \tag{S12}$$

$P(\alpha_i|\Psi)$ is the probability that Ψ codes for codon α_i and is defined as

$$P(\alpha_i|\Psi) = \sum_{c \in C_{\alpha_i}} P(c|\Psi_i) \tag{S13}$$

C_{α_i} is the set of nucleotide triplets coding for codon α_i, Ψ_i is the subsequence of Ψ corresponding to the ith codon, and $P(c|\Psi_i)$ is the probability of this subsequence coding for triplet c. Like ECAI, $P(\alpha|\Psi)$ can be computed efficiently by computing the probability of each codon subsequence independently.

We can then combine these two quantities to formulate a loss function for mRNA design over a distribution of sequences. Given an arbitrary metric of mRNA stability O where higher numbers represent greater stability, we represent the loss function of a distribution of sequences Ψ as

$$-\mathcal{L}(\Psi) = O(\Psi) \cdot \phi_C(\mathrm{ECAI}(\Psi)) \cdot \phi_P(P(\alpha|\Psi)) \tag{S14}$$

where ϕ_C and ϕ_P are activation functions that enforce minimum values of $\mathrm{ECAI}(\Psi)$ and $P(\alpha|\Psi)$, respectively. Both ϕ_C and ϕ_P "activate" if their values are below a threshold, which increases the loss. There are many choices of activation function; in our experiment we used a Leaky ReLU and a quadratic spline, see "Optimization Details" for more information.

We use take the product of loss terms instead of the sum to account for the different scales of each term. This is analogous to using a geometric mean instead of an arithmetic mean. The negative sign indicates the formulation of the optimization problem as a minimization problem. In our experiments we use $O(\Psi) = \mathcal{Z}_\Psi = \sum_\pi p(\pi|\Psi) \cdot Z_\pi$.

Since we can compute $O(\Psi) = \mathcal{Z}_\Psi$ using differentiable folding, Equation S14 is continuous and differentiable and we can directly optimize Ψ via gradient descent. Given an optimized Ψ, we obtain a final optimized nucleotide sequence by sampling discrete sequences from Ψ and choosing the sampled sequence that maximizes stability while satisfying the minimum value of CAI and coding for α as per our initial objective Equation S15.

Optimization Details

In Table 2, we report the optimized ensemble free energies via LinearDesign [Zhang et al., 2023] and our method for five benchmark protein sequences. To generate results via LinearDesign for unconstrained CAI, we ran the publicly available executable with default settings and $\lambda = 0$. To find the optimal coding sequence subject to CAI ≥ 0.8 with LinearDesign, we performed binary search over λ to find the minimal λ (and therefore the minimum MFE) corresponding to a sequence satisfying the CAI constraint.

For the loss terms ϕ_C and ϕ_P, we use two different activation functions and thresholds. For ϕ_C, we use a Leaky ReLU with with a slope of 1.0 for ECAI < 0.8 and a slope of 0.05 for ECAI ≥ 0.8. For ϕ_P, we use a quadratic spline with a quadratic coefficient of 50 for $P(\alpha|\Psi) < 0.95$ and a linear slope of 0.025 for $P(\alpha|\Psi) \geq 0.95$.

All optimizations via our method were performed in JAX [Bradbury et al., 2018] on an 80 GB NVIDIA A100. To ensure that the continuous sequence is properly normalised, in practice we optimize a set of logits that are normalised via a softmax operator at each optimization step. Each optimization is performed in three steps:

1. Initialization of logits
2. Neural network pretraining
3. Sequence optimization

Logits are initialisized via the LinearDesign solution. For a nucleotide sequence of length n, the logits are initialised as $10 \times \mathrm{onehot}(\mathrm{seq}_{LD}) + 10$ where seq_{LD} is the LinearDesign solution and $\mathrm{onehot}(\mathrm{seq}_{LD})$ is the $n \times 4$ one-hotted array corresponding to this discrete sequence.

Next, we pretrain a fully-connected neural network to predict this initial logits given a fixed random seed. We use a network with 6 layers, each consisting of 4000 features, and Leaky ReLU as an activation function. We define the pretraining loss function as the MSD between the predicted logits and the target initial logits. Pretraining is performed via 250 iterations with an Adam optimizer and a learning rate of 0.0001.

Finally, we apply gradient descent to the parameters of this pretrained network to predict logits that minimise our target objective function for mRNA. Each optimization was run for 5000 iterations (or until convergence) with a Lamb optimizer and a learning rate of 10^{-5}.

Note that we tried to match the thermodynamic model used by LinearDesign [Zhang et al., 2023]. Since their method is closed source, it is hard to be certain. However, they appear to use the same model as ViennaRNA [Lorenz et al., 2011] with the `-d0` flag enabled, which does not count dangling ends.

Relationship to Previous Work

Terai et al. [2016] appear to be the first to propose stability-CAI optimization. They proposed to minimize $\Omega(\pi|\alpha) = \mathrm{MFE}(\pi) \times \mathrm{CAI}(\pi|\alpha)^\lambda$ where $\mathrm{MFE}(\pi)$ denotes the *minimum free energy* (MFE) for a sequence π, which is the free energy of the most stable structure in the ensemble [Zuker and Stiegler, 1981]. Note that λ is an arbitrary weighting factor. Terai et al. [2016] proposed an algorithm to find a sequence with minimum MFE, but it could not simultaneously optimize CAI [Terai et al., 2016]. Later, Zhang *et al.* solved the problem [Zhang et al., 2023]. They proposed LinearDesign, which minimizes $\Omega(\pi|\alpha) = \mathrm{MFE}(\pi) - \lambda \log(\mathrm{CAI}(\pi|\alpha))$. LinearDesign represented a breakthrough, as it was the first algorithm to effectively do stability-CAI optimization.

Other stability-CAI objectives have been proposed. Wayment-Steele et al. [2021] proposed *average unpaired probability* (AUP), an improved measure of stability, and Ribotree, an algorithm for optimizing mRNA using a modified Monte Carlo tree search. AUP was experimentally determined to be a better measure of stability than MFE [Leppek et al., 2022]. This is intuitive, since MFE only considers a single structure, but AUP considers the entire ensemble. Wayment-Steele et al. [2021] also tried optimizing EFE, which is the free energy of the entire ensemble rather than just the most stable structure as in MFE, and is related to the stability measure we apply in our work via Equation S9.

In the previously mentioned approaches a weighting factor λ is used to balance stability and CAI. Instead, we use set a threshold for CAI. That is, we optimize a sequence π to

maximize stability subject to the condition that its CAI is at least τ:

$$\Omega(\pi|\alpha) = \begin{cases} Z_\pi & \text{if } \mathrm{CAI}(\pi|\alpha) \geq \tau \\ -\infty & \text{otherwise} \end{cases} \tag{S15}$$

For our purposes, the mRNA design problem is to find an mRNA π that optimizes Equation S15 given an amino acid sequence α. The methods we present can likely be adapted to more complex objectives. These might include other measures of stability, such as AUP. They might also include other considerations relevant to a vaccine designer, such as 5'-leader region optimization [Mauger et al., 2019], or uridine depletion [Vaidyanathan et al., 2018]. However, Equation S15 is used here as it is at least as powerful as existing widely-used objectives and is simple enough for a concise presentation.

Comparison to Dai et al.

During the preparation of this manuscript a preprint by Dai et al. [2024] was published that optimizes mRNA sequences similarly, i.e. via differentiable folding. There is overlap between our work, but also some major differences. Both methods optimize the stability of mRNA sequences measured by expected partition function using a differentiable folding algorithm. There are several differences.

First, our method builds upon the differentiable folding algorithm of Matthies et al. [2023]. Dai et al. [2024] do not use the same algorithm but instead implement a new folding algorithm based on LinearPartition [Zhang et al., 2020] and LinearDesign [Zhang et al., 2023]. The upshot of using different folding algorithms is that Dai et al. optimize an approximate partition function, whereas we optimize the complete partition function. This makes their method use fewer resources, but at the cost of lower quality gradients. In addition, our algorithm runs on GPU but the Dai et al. method is CPU-only. These folding algorithms are conceptually different as well as practically different. We operate at the level of nucleotides and use Equation S13 to enforce valid codon sequences, but Dai et al. operate at the level of codons.

Second, Dai et al. do not incorporate differentiable folding as a module in a larger deep learning architecture. In contrast, one of our major contributions is building a deep learning pipeline to optimize sequences. To achieve good results, Dai et al. used multiple initializations, whereas we only used a single run to generate our results.

Finally, we optimize a stability-CAI objective using the expected CAI loss term described by Equation S11. Dai et al. only optimize stability.

Puzzle ID	Direct	Neural Network	Answer 1	Answer 2
1	9.95×10^{-1}	9.95×10^{-1}	4.17×10^{-1}	9.32×10^{-1}
2	8.28×10^{-1}	9.39×10^{-1}	8.58×10^{-3}	6.85×10^{-1}
3	9.81×10^{-1}	9.87×10^{-1}	3.88×10^{-1}	7.74×10^{-1}
4	7.26×10^{-2}	6.53×10^{-1}	4.52×10^{-1}	2.99×10^{-1}
5	8.93×10^{-1}	8.21×10^{-1}	NA	NA
6	1.81×10^{-8}	1.24×10^{-1}	1.29×10^{-6}	2.00×10^{-12}
7	1.92×10^{-1}	3.04×10^{-1}	6.13×10^{-11}	5.78×10^{-3}
8	9.79×10^{-1}	9.79×10^{-1}	1.04×10^{-1}	1.37×10^{-1}
9	4.11×10^{-8}	9.74×10^{-2}	8.27×10^{-2}	2.17×10^{-1}
10	7.74×10^{-1}	9.46×10^{-1}	5.15×10^{-2}	1.27×10^{-1}
11	9.61×10^{-1}	9.76×10^{-1}	6.09×10^{-1}	6.23×10^{-1}
12	4.90×10^{-1}	8.00×10^{-1}	6.01×10^{-6}	5.61×10^{-5}
13	9.73×10^{-1}	9.73×10^{-1}	9.27×10^{-1}	9.68×10^{-1}
14	4.07×10^{-8}	4.58×10^{-7}	8.79×10^{-7}	2.41×10^{-8}
15	1.27×10^{-5}	2.60×10^{-5}	4.12×10^{-4}	1.05×10^{-3}
16	1.25×10^{-8}	4.55×10^{-10}	1.50×10^{-6}	2.24×10^{-6}
17	5.87×10^{-8}	5.24×10^{-8}	3.92×10^{-5}	2.16×10^{-7}
18	9.68×10^{-1}	9.79×10^{-1}	2.87×10^{-2}	2.16×10^{-1}
19	6.60×10^{-1}	7.74×10^{-1}	6.99×10^{-1}	4.66×10^{-2}
20	2.14×10^{-1}	6.17×10^{-1}	3.45×10^{-1}	6.00×10^{-1}
21	4.34×10^{-8}	7.82×10^{-2}	3.29×10^{-4}	1.50×10^{-1}
22	1.00×10^{0}	9.58×10^{-1}	1.00×10^{0}	9.68×10^{-1}
23	1.60×10^{-1}	2.71×10^{-1}	9.37×10^{-4}	6.76×10^{-3}
24	6.90×10^{-3}	6.17×10^{-1}	4.39×10^{-1}	7.71×10^{-2}
25	3.67×10^{-1}	3.71×10^{-1}	1.07×10^{-3}	1.52×10^{-5}
26	9.19×10^{-1}	9.88×10^{-1}	1.41×10^{-1}	1.45×10^{-1}
27	4.27×10^{-1}	6.64×10^{-1}	1.64×10^{-2}	4.50×10^{-2}
28	2.78×10^{-1}	1.87×10^{-1}	6.00×10^{-5}	9.25×10^{-4}
29	2.39×10^{-2}	5.03×10^{-1}	7.29×10^{-5}	3.70×10^{-9}
30	9.14×10^{-1}	9.78×10^{-1}	7.16×10^{-2}	1.37×10^{-1}
31	8.55×10^{-1}	9.55×10^{-1}	1.69×10^{-1}	4.69×10^{-2}
32	2.46×10^{-1}	8.26×10^{-1}	6.86×10^{-1}	1.26×10^{-4}
33	8.15×10^{-1}	8.92×10^{-1}	7.61×10^{-1}	7.13×10^{-1}
34	6.45×10^{-5}	2.07×10^{-3}	1.31×10^{-5}	1.54×10^{-5}
35	9.32×10^{-17}	4.38×10^{-18}	2.72×10^{-20}	2.67×10^{-22}
36	4.54×10^{-6}	1.84×10^{-5}	5.36×10^{-11}	4.14×10^{-12}
37	4.66×10^{-8}	1.90×10^{-8}	9.18×10^{-8}	1.04×10^{-9}
38	4.78×10^{-9}	1.18×10^{-6}	7.74×10^{-14}	3.09×10^{-15}
39	2.06×10^{-1}	7.06×10^{-1}	7.87×10^{-6}	6.75×10^{-1}
40	3.14×10^{-1}	9.53×10^{-1}	1.84×10^{-2}	5.16×10^{-3}
41	4.07×10^{-6}	1.51×10^{-3}	1.16×10^{-6}	8.42×10^{-8}
42	9.22×10^{-5}	1.46×10^{-5}	3.12×10^{-3}	1.81×10^{-2}
43	2.33×10^{-1}	8.24×10^{-1}	1.41×10^{-1}	3.83×10^{-1}
44	6.85×10^{-2}	1.82×10^{-1}	2.78×10^{-5}	7.90×10^{-3}
45	3.30×10^{-4}	9.64×10^{-1}	6.51×10^{-1}	1.70×10^{-1}
46	4.03×10^{-12}	9.11×10^{-4}	1.69×10^{-6}	2.36×10^{-5}
47	3.42×10^{-3}	1.04×10^{-2}	2.13×10^{-5}	1.19×10^{-4}
48	5.99×10^{-4}	8.48×10^{-4}	5.47×10^{-4}	9.84×10^{-3}
49	7.52×10^{-3}	9.33×10^{-2}	1.78×10^{-2}	1.23×10^{-4}
50	7.82×10^{-13}	6.73×10^{-14}	4.12×10^{-11}	6.73×10^{-11}
51	3.23×10^{-8}	3.21×10^{-2}	1.26×10^{-7}	2.41×10^{-14}
52	9.05×10^{-10}	6.25×10^{-8}	3.38×10^{-8}	6.79×10^{-8}
53	1.41×10^{-6}	8.62×10^{-4}	4.99×10^{-1}	1.07×10^{-1}
54	5.04×10^{-1}	8.83×10^{-1}	4.03×10^{-1}	1.26×10^{-2}
55	2.63×10^{-5}	1.76×10^{-4}	3.60×10^{-4}	1.81×10^{-2}
56	2.61×10^{-1}	4.31×10^{-1}	3.58×10^{-21}	2.24×10^{-18}
57	1.21×10^{-14}	7.47×10^{-13}	2.21×10^{-17}	1.99×10^{-17}
58	5.45×10^{-3}	7.20×10^{-2}	3.87×10^{-7}	4.23×10^{-3}
59	2.00×10^{-2}	8.88×10^{-2}	1.51×10^{-4}	3.82×10^{-4}
60	7.11×10^{-4}	2.93×10^{-3}	9.11×10^{-3}	1.03×10^{-2}
61	4.77×10^{-12}	1.46×10^{-9}	7.90×10^{-11}	3.63×10^{-11}

62	1.41×10^{-2}	$\underline{1.02 \times 10^{-1}}$	2.66×10^{-5}	1.69×10^{-6}
63	5.37×10^{-2}	$\underline{6.58 \times 10^{-2}}$	1.34×10^{-24}	7.46×10^{-41}
64	9.23×10^{-5}	1.33×10^{-3}	$\underline{2.04 \times 10^{-2}}$	5.91×10^{-3}
65	3.05×10^{-5}	$\underline{5.90 \times 10^{-4}}$	1.61×10^{-4}	1.83×10^{-4}
66	1.94×10^{-3}	$\underline{3.44 \times 10^{-2}}$	6.17×10^{-4}	2.31×10^{-4}
67	1.11×10^{-2}	$\underline{1.42 \times 10^{-1}}$	1.52×10^{-2}	5.49×10^{-2}
68	8.78×10^{-15}	$\underline{2.6 \times 10^{-14}}$	3.16×10^{-32}	1.19×10^{-25}
69	2.49×10^{-3}	$\underline{6.72 \times 10^{-3}}$	6.81×10^{-9}	1.80×10^{-16}
70	$\underline{4.27 \times 10^{-1}}$	3.04×10^{-2}	7.88×10^{-14}	6.29×10^{-21}
71	1.79×10^{-4}	$\underline{1.20 \times 10^{-1}}$	1.47×10^{-5}	1.43×10^{-5}
72	4.69×10^{-3}	$\underline{1.80 \times 10^{-1}}$	1.71×10^{-2}	2.90×10^{-2}
73	9.79×10^{-66}	4.36×10^{-35}	1.06×10^{-31}	$\underline{9.96 \times 10^{-31}}$
74	3.02×10^{-3}	$\underline{3.57 \times 10^{-2}}$	2.39×10^{-112}	2.92×10^{-110}
75	5.57×10^{-2}	$\underline{5.54 \times 10^{-1}}$	1.12×10^{-2}	1.73×10^{-2}
76	4.03×10^{-45}	3.09×10^{-53}	$\underline{4.01 \times 10^{-44}}$	8.49×10^{-55}
77	1.71×10^{-4}	$\underline{9.57 \times 10^{-2}}$	2.49×10^{-4}	4.63×10^{-5}
78	2.94×10^{-22}	$\underline{4.15 \times 10^{-16}}$	1.52×10^{-73}	4.29×10^{-70}
79	2.23×10^{-3}	9.66×10^{-4}	3.76×10^{-5}	$\underline{5.41 \times 10^{-3}}$
80	3.06×10^{-3}	$\underline{1.48 \times 10^{-2}}$	5.18×10^{-8}	6.60×10^{-11}
81	7.29×10^{-15}	4.59×10^{-14}	9.76×10^{-18}	$\underline{1.27 \times 10^{-13}}$
82	4.09×10^{-3}	$\underline{2.36 \times 10^{-1}}$	2.70×10^{-13}	3.80×10^{-10}
83	8.51×10^{-19}	$\underline{2.47 \times 10^{-16}}$	7.54×10^{-26}	6.13×10^{-29}
84	3.39×10^{-6}	4.18×10^{-4}	$\underline{1.25 \times 10^{-3}}$	2.66×10^{-5}
85	2.37×10^{-40}	5.43×10^{-33}	$\underline{3.41 \times 10^{-13}}$	2.45×10^{-13}
86	1.01×10^{-64}	1.92×10^{-65}	$\underline{5.44 \times 10^{-46}}$	2.75×10^{-57}
87	8.61×10^{-9}	1.45×10^{-8}	$\underline{7.53 \times 10^{-8}}$	1.22×10^{-9}
88	2.28×10^{-2}	$\underline{3.89 \times 10^{-2}}$	1.38×10^{-2}	1.24×10^{-2}
89	2.24×10^{-9}	$\underline{2.96 \times 10^{-5}}$	6.99×10^{-16}	2.25×10^{-15}
90	4.38×10^{-40}	$\underline{7.77 \times 10^{-32}}$	5.36×10^{-41}	3.08×10^{-42}
91	4.89×10^{-31}	$\underline{1.66 \times 10^{-23}}$	4.19×10^{-53}	5.88×10^{-54}
92	1.19×10^{-19}	4.83×10^{-22}	1.35×10^{-16}	$\underline{1.25 \times 10^{-15}}$
93	4.58×10^{-7}	$\underline{4.05 \times 10^{-3}}$	7.62×10^{-10}	1.60×10^{-9}
94	1.31×10^{-41}	$\underline{4.01 \times 10^{-24}}$	2.39×10^{-38}	6.18×10^{-44}
95	6.46×10^{-6}	5.82×10^{-5}	$\underline{1.43 \times 10^{-4}}$	1.75×10^{-6}
96	9.26×10^{-54}	$\underline{5.1 \times 10^{-53}}$	3.17×10^{-102}	1.59×10^{-102}
97	3.67×10^{-22}	$\underline{1.02 \times 10^{-16}}$	1.65×10^{-19}	2.12×10^{-19}
98	1.29×10^{-13}	6.72×10^{-10}	1.01×10^{-11}	$\underline{1.24 \times 10^{-8}}$
99	1.53×10^{-43}	$\underline{2.74 \times 10^{-35}}$	2.05×10^{-43}	1.22×10^{-41}
100	$\underline{1.04 \times 10^{-30}}$	1.42×10^{-31}	NA	NA

Table S1: Probabilities of optimized sequences for the entire Eterna100 dataset using our default treatment of coaxial stacks, terminal mismatches, and dangling ends (corresponding to the `d0` option in ViennaRNA). "Direct" and "Neural Network" correspond to sequences optimized via our method with and without overparameterization with a neural network, respectively. For each optimized sequence, 500 iterations of gradient descent were performed with a Lamb optimizer. A learning rate of 0.01 and 0.0001 was used for "Direct" and "Neural Network", respectively. These learning rates were determined via a hyperparameter search over learning rates at varying orders of magnitude. "Answer 1" and "Answer 2" correspond to the provided solutions in the Eterna100 dataset. A value of "NA" indicates that no solution was provided in the original dataset. Note that the Eterna100 was not designed with respect to the `d0` recursions so these solutions are not expected to be optimal, but we provide their performance on `d0` for reference. For each puzzle, the underlined value represents the solution with the highest probability.

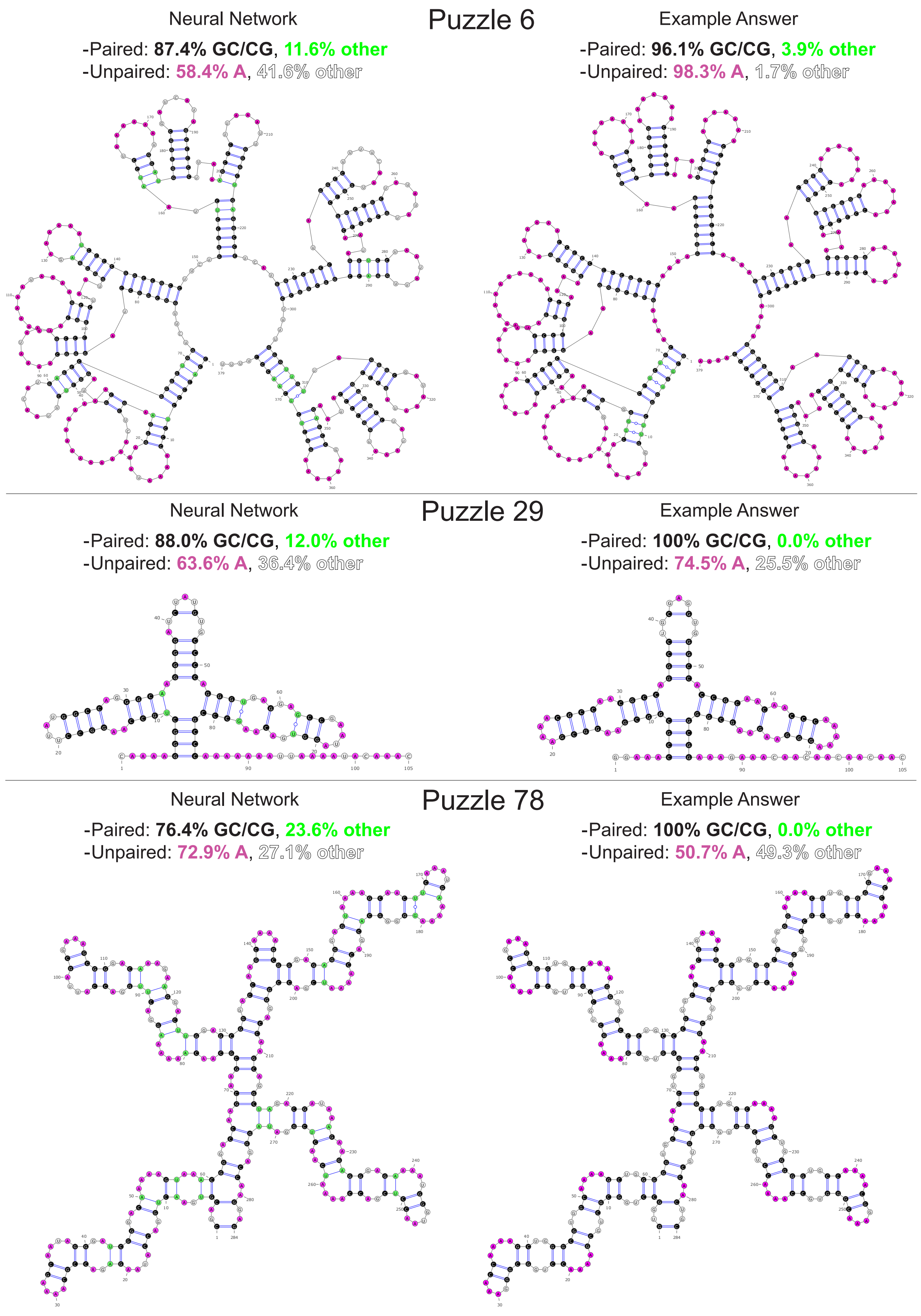

Fig. S1. A comparison of sequence motifs between solutions optimized via our method vs. example solutions in the Eterna100 dataset. For each puzzle, the target structure is colored according to the sequence. Base pairs are colored green if they are GC or CG and black otherwise. Unpaired nucleotides are colored pink if they are an A and white otherwise. The example answers correspond to solutions 2, 1, and 2 for puzzles 6, 29, and 78, respectively. Illustrations were generated with VARNA [Darty et al., 2009]. For all puzzles, our solution outperforms the provided solution (see Table S1).

Metric	Direct	Neural Network	Answer 1	Answer 2
% GC/CG Pairs	84.2	90.3	59.4	59.3
% Unpaired A	61.4	65.1	80.6	79.2

Table S2. Average probability of sequence motifs for the solutions provided in Table S1. For each puzzle, we compute the percentage of paired nucleotides that are GC or CG and the percentage of unpaired nucleotides that are A. This represents a measurement of agreement with a trivial design algorithm in which paired nucleotides would be assigned in this fashion. "% GC/CG Pairs" and "% Unpaired A" represent these quantities averaged over all puzzles. "Direct" and "Neural Network" correspond to sequences optimized via our method with and without overparameterization with a neural network, respectively. "Answer 1" and "Answer 2" correspond to the provided solutions in the Eterna100 dataset.

Type	Optimization	Sequence Length	Time per Iteration (s)	Num. Iterations	Total Time (h)
mRNA	MEV, unconstrained	141	0.675	3203	0.61
	MEV, CAI $\geq$ 0.8	141	0.675	970	0.19
	Mini-GFP	330	4.04	2549	2.9
	Mini-GFP, CAI $\geq$ 0.8	330	4.03	0	0.0
	Nanoluciferase	663	15.6	3266	14
	Nanoluciferase, CAI $\geq$ 0.8	663	12.5	1509	5.3
	spike RBD	630	11.6	2585	8.3
	spike RBD, CAI $\geq$ 0.8	630	11.1	563	1.7
	eGFP + degron	852	20.2	1041	5.9
	eGFP + degron, CAI $\geq$ 0.8	852	20.2	610	3.4
Structure	Puzzle 8, No Network	12	2.65×10^{-3}	500	0.014
	Puzzle 8, Neural Network	12	4.69×10^{-3}	500	0.014
	Puzzle 63, No Network	400	5.07	500	0.71
	Puzzle 63, Neural Network	400	5.82	500	0.82

Table S3. Total clock time for example optimizations. We include all mRNA optimizations from Table 2 and two example structural optimizations from Table S1, representing the minimum and maximum sequence length in the Eterna100. "Sequence Length" is the length of the corresponding nucleotide sequence. "Time per Iteration" is the average time per gradient update in seconds *after* just-in-time compilation via JAX (which poses an additional upfront cost). "Num Iterations" is the number of iterations until convergence for the mRNA optimizations, and the total number of iterations (500) for the structural optimizations. The number of iterations for "Mini-GFP, CAI $\geq$ 0.8" is 0 because our method did not improve upon the LinearDesign solution. mRNA optimizations were run for up to 5000 iterations. "Total Time" is the total wall clock time in hours for the listed number of iterations, including compilation time. Note that a single gradient update for structural optimization requires *two* passes through differentiable folding to compute both the numerator and the denominator in Equation (S4).

References

J. Bradbury, R. Frostig, P. Hawkins, M. J. Johnson, C. Leary, D. Maclaurin, G. Necula, A. Paszke, J. VanderPlas, S. Wanderman-Milne, and Q. Zhang. JAX: composable transformations of Python+NumPy programs, 2018. URL http://github.com/google/jax.

N. Dai, W. Y. Tang, T. Zhou, D. H. Mathews, and L. Huang. Messenger and non-coding rna design via expected partition function and continuous optimization. *arXiv preprint arXiv:2401.00037*, 2024.

K. Darty, A. Denise, and Y. Ponty. VARNA: Interactive drawing and editing of the RNA secondary structure. *Bioinformatics*, 25(15):1974, 2009.

K. Leppek, G. W. Byeon, W. Kladwang, H. K. Wayment-Steele, C. H. Kerr, A. F. Xu, D. S. Kim, V. V. Topkar, C. Choe, D. Rothschild, et al. Combinatorial optimization of mrna structure, stability, and translation for rna-based therapeutics. *Nature communications*, 13(1):1536, 2022.

R. Lorenz, S. H. Bernhart, C. Höner zu Siederdissen, H. Tafer, C. Flamm, P. F. Stadler, and I. L. Hofacker. ViennaRNA package 2.0. *Algorithms for molecular biology*, 6(1):1–14, 2011.

M. C. Matthies, R. Krueger, A. E. Torda, and M. Ward. Differentiable partition function calculation for RNA. *Nucleic Acids Research*, page gkad1168, 12 2023. ISSN 0305-1048. doi: 10.1093/nar/gkad1168. URL https://doi.org/10.1093/nar/gkad1168.

D. M. Mauger, B. J. Cabral, V. Presnyak, S. V. Su, D. W. Reid, B. Goodman, K. Link, N. Khatwani, J. Reynders, M. J. Moore, et al. mrna structure regulates protein expression through changes in functional half-life. *Proceedings of the National Academy of Sciences*, 116(48):24075–24083, 2019.

J. S. McCaskill. The equilibrium partition function and base pair binding probabilities for RNA secondary structure. *Biopolymers: Original Research on Biomolecules*, 29(6-7):1105–1119, 1990.

P. M. Sharp and W.-H. Li. The codon adaptation index-a measure of directional synonymous codon usage bias, and its potential applications. *Nucleic acids research*, 15(3):1281–1295, 1987.

G. Terai, S. Kamegai, and K. Asai. Cdsfold: an algorithm for designing a protein-coding sequence with the most stable secondary structure. *Bioinformatics*, 32(6):828–834, 2016.

S. Vaidyanathan, K. T. Azizian, A. A. Haque, J. M. Henderson, A. Hendel, S. Shore, J. S. Antony, R. I. Hogrefe, M. S. Kormann, M. H. Porteus, et al. Uridine depletion and chemical modification increase cas9 mrna activity and reduce immunogenicity without hplc purification. *Molecular Therapy-Nucleic Acids*, 12:530–542, 2018.

H. K. Wayment-Steele, D. S. Kim, C. A. Choe, J. J. Nicol, R. Wellington-Oguri, A. M. Watkins, R. A. Parra Sperberg, P.-S. Huang, E. Participants, and R. Das. Theoretical basis for stabilizing messenger rna through secondary structure design. *Nucleic acids research*, 49(18):10604–10617, 2021.

H. Zhang, L. Zhang, D. H. Mathews, and L. Huang. Linearpartition: linear-time approximation of rna folding partition function and base-pairing probabilities. *Bioinformatics*, 36(Supplement_1):i258–i267, 2020.

H. Zhang, L. Zhang, A. Lin, C. Xu, Z. Li, K. Liu, B. Liu, X. Ma, F. Zhao, H. Jiang, et al. Algorithm for optimized mrna design improves stability and immunogenicity. *Nature*, pages 1–3, 2023.

M. Zuker and P. Stiegler. Optimal computer folding of large rna sequences using thermodynamics and auxiliary information. *Nucleic acids research*, 9(1):133–148, 1981.
